# A Hybrid and Comparative Machine Learning Framework for Predicting Gestational Diabetes Mellitus via a Prospective Case-Control Design: A Pilot Study Integrating Periodontal Health and Hematological Inflammatory Markers

**DOI:** 10.3390/metabo16090617

**Published:** 2026-08-27

**Authors:** İsa Temur, Mehmet Özsan, Katibe Tuğçe Temur, Andaç Batur Çolak

**Affiliations:** 1 Department of Obstetrics and Gynecology, Faculty of Medicine, Niğde Ömer Halisdemir University, 51240 Nigde, Türkiye; isatemur@ohu.edu.tr; 2 Department of Physiology, Faculty of Medicine, Niğde Ömer Halisdemir University, 51240 Nigde, Türkiye; 3 Department of Oral and Maxillofacial Radiology, Faculty of Dentistry, Niğde Ömer Halisdemir University, 51240 Nigde, Türkiye; tugcetemur@ohu.edu.tr; 4 Department of Information Systems and Technologies, Faculty of Computer and Information Sciences, Niğde Ömer Halisdemir University, 51240 Nigde, Türkiye; bcolak@ohu.edu.tr

**Keywords:** gestational diabetes mellitus, machine learning, artificial neural network, periodontal disease, inflammatory biomarkers, complete blood count, precision medicine, pregnancy, risk prediction

## Abstract

**Highlights:**

**What are the main findings?**

- A hybrid machine learning framework successfully integrated periodontal and hematological biomarkers for accurate prediction of gestational diabetes mellitus.
- Bayesian-regularized artificial neural networks achieved superior predictive performance and robustness compared with a transformer-enhanced physics-informed neural network.

**What are the implications of the main findings?**

- Routine clinical and periodontal data can be combined to develop accurate, low-cost, and non-invasive screening strategies for gestational diabetes.
- The proposed framework has the potential to facilitate precision obstetrics by enabling earlier identification and management of high-risk pregnancies.

**Abstract:**

**Background/Objectives**: This pilot study aimed to develop and compare advanced machine learning models, specifically a Bayesian-regularized artificial neural network (ANN) and a transformer-enhanced physics-informed neural network (PINN), by integrating periodontal health indices and hematological inflammatory markers for the prediction of GDM. **Methods**: Utilizing a prospective case–control study design, a clinical dataset comprising 80 pregnant women (40 with GDM and 40 healthy controls) was evaluated to develop and compare advanced machine learning models. Clinical, periodontal, and complete blood count-derived inflammatory parameters were integrated into two predictive models: a Bayesian regularization-based artificial neural network (ANN) and a transformer-enhanced physics-informed neural network (PINN). Model performance was evaluated using the coefficient of determination (R^2^), mean squared error (MSE), root mean squared error (RMSE), mean absolute error (MAE), and residual error analyses. **Results**: The ANN demonstrated superior predictive performance, achieving an R^2^ of 0.9872 and an MSE of 3.38 × 10^−3^, compared with an R^2^ of 0.9833 for the PINN model. Residual analysis showed that the ANN provided greater prediction stability, with a mean deviation of 0.5691 and a standard deviation of 3.6400. The findings demonstrate that the integrated analysis of periodontal and hematological markers through advanced neural architectures, particularly the Bayesian-regularized ANN, provides a high-fidelity diagnostic signal for GDM prediction. This integrated feature set, when processed by the proposed machine learning framework, enables the identification of complex biological patterns with remarkable precision. **Conclusions**: The proposed machine learning framework provides an accurate, non-invasive, and clinically applicable approach for predicting GDM. Integrating routinely available periodontal and hematological data may improve early risk stratification and support personalized prenatal care. Further validation in larger and more diverse populations is warranted before clinical implementation.

## 1. Introduction

Gestational Diabetes Mellitus (GDM) is one of the most common metabolic complications of pregnancy and remains a major cause of maternal and fetal morbidity and mortality. GDM is not merely a temporary metabolic disorder limited to pregnancy. It is also associated with an increased long-term risk of type 2 diabetes mellitus, cardiovascular disease, and chronic inflammatory conditions in both the mother and the child [1,2,3]. Recent studies have demonstrated that systemic inflammation, immune dysregulation, and metabolic disturbances play a central role in the pathogenesis of GDM. This has increased interest in reliable biomarkers for early diagnosis and risk assessment.

Periodontal Disease is a chronic inflammatory condition affecting the gingiva, periodontal ligament, cementum, and alveolar bone. It is characterized by progressive tissue destruction and alveolar bone loss [1]. In recent years, periodontal inflammation has been linked not only to local oral damage but also to several systemic diseases, particularly diabetes mellitus and pregnancy-related complications [2,3]. Hormonal and immunological changes during pregnancy increase the susceptibility of periodontal tissues to inflammation. This process may elevate the systemic inflammatory burden and contribute to the development of insulin resistance. Several studies have reported significant associations between periodontal disease and adverse obstetric outcomes such as preterm birth, low birth weight, and GDM [4].

On the other hand, although there are findings in the literature supporting a relationship between periodontal disease and GDM, the results are not entirely consistent. A recent meta-analysis showed that case–control and cohort studies were associated with an increased risk of GDM related to periodontal disease, while cross-sectional studies found no significant association. However, due to the heterogeneity and methodological differences among the studies, it has been emphasized that this relationship needs to be supported by further studies [5]. Despite this inconsistency in the literature, several biological mechanisms have been proposed to explain a plausible link between periodontal inflammation and GDM. Pregnancy is a unique physiological condition accompanied by marked hormonal, vascular, and immunological changes. Elevated estrogen and progesterone levels increase vascular permeability in gingival tissues, leading to an exaggerated inflammatory response against bacterial biofilms [6]. Increased levels of systemic inflammatory markers such as interleukin-6 (IL-6) and C-reactive protein (CRP) have been reported in pregnant women with periodontal disease. These markers have also been associated with insulin resistance and impaired glucose metabolism [6,7]. Such findings suggest that periodontal inflammation may contribute to the systemic inflammatory mechanisms involved in the development of GDM.

Inflammatory markers derived from complete blood count (CBC) parameters have recently attracted attention as practical, inexpensive, and easily accessible biomarkers for evaluating systemic inflammation during pregnancy [8,9]. Hematological indices such as the neutrophil-to-lymphocyte ratio (NLR), platelet-to-lymphocyte ratio (PLR), monocyte-to-lymphocyte ratio (MLR), and platelet indices may reflect both metabolic dysregulation and inflammatory activity. Because these parameters can be obtained routinely in clinical practice, they may provide valuable information regarding the inflammatory processes associated with periodontal disease and GDM.

In our previously published case–control study, pregnant women diagnosed with GDM showed significantly higher plaque index (PI), gingival index (GI), probing depth (PD), and DMFT scores compared with healthy pregnant women. Significant associations were also identified between periodontal parameters and hematological inflammatory markers [10]. These findings support the close relationship between oral inflammation and systemic inflammatory dysregulation and highlight the potential role of periodontal health in the pathophysiology of GDM.

Most previous studies investigating the relationship between periodontal disease, inflammatory markers, and GDM have relied on conventional statistical methods. However, these approaches may be insufficient for identifying the complex and nonlinear interactions among metabolic, hematological, behavioral, and periodontal variables. Since gestational diabetes is a multifactorial disorder influenced by several interconnected biological mechanisms, advanced computational approaches may provide greater predictive power than conventional regression-based analyses [11,12,13,14,15].

In recent years, artificial intelligence (AI) and machine learning (ML) algorithms have been increasingly used to predict pregnancy complications such as gestational diabetes, preeclampsia, and preterm birth [11,12,13,14,15]. Physiology-based artificial neural network (ANN) models that simultaneously evaluate hematological, dental, and periodontal parameters have achieved high accuracy rates in predicting preterm birth and low birth weight risk [16]. ANN has become particularly important in this field because they can analyze complex relationships among multiple biological variables at the same time [13,14,15]. Previous studies have shown that ANN-based models may outperform traditional statistical methods in identifying hidden patterns within clinical datasets and improving the prediction accuracy of metabolic diseases [11,12,13]. The ability of ANN to evaluate multivariable biological systems together offers major advantages for the early prediction of multifactorial diseases.

AI-supported approaches have also become increasingly common in the evaluation of Periodontal Disease. Deep learning-based models have produced promising results in grading periodontal destruction, assessing inflammatory processes, and analyzing oral-systemic relationships [17]. However, to the best of our knowledge, no previous study has comprehensively evaluated periodontal indices, CBC derived inflammatory markers, maternal clinical variables, and behavioral parameters within a single ANN-based predictive model for GDM.

The objective of this study was to develop and compare advanced machine learning models, namely a Bayesian-regularized ANN and a transformer-enhanced PINN, by integrating periodontal indices and hematological inflammatory markers. It was hypothesized that the ANN model would demonstrate superior predictive performance in identifying GDM risk compared to the hybrid PINN architecture, thereby providing a robust and non-invasive screening tool for early risk stratification.

## 2. Materials and Methods

### 2.1. Study Design and Ethical Approval

A prospective case–control design was utilized to assess the predictive capacity of integrated periodontal and hematological markers. The study was conducted at the Department of Obstetrics and Gynecology, Niğde Ömer Halisdemir University Faculty of Medicine between September 2022 and April 2023. The study protocol was approved by the university’s Non-Interventional Clinical Research Ethics Committee (Decision No.: 2022/72). All procedures were performed in accordance with the principles of the Declaration of Helsinki.

### 2.2. Participants and Study Groups

A balanced dataset consisting of 80 participants was created for the training and testing phases of the model.

Case Group (*n* = 40): Pregnant women diagnosed with GDM between the 24th and 28th weeks of pregnancy according to IADPSG criteria. Diagnosis was based on the 75 g oral glucose tolerance test (OGTT): fasting glucose ≥ 92 mg/dL, 1-h glucose ≥ 180 mg/dL, or 2-h glucose ≥ 153 mg/dL.

Control Group (*n* = 40): Healthy pregnant women at the same gestational weeks without GDM or pregnancy complications.

Women aged 18–35 years with singleton pregnancies were included in the study. To maintain dataset homogeneity, participants with factors that could affect inflammatory outcomes were excluded. These factors included antibiotic or mouthwash use within the previous six months, periodontal treatment within the previous three months, active infection, fewer than 20 teeth, and a history of systemic disease.

### 2.3. Sample Size and Power Analysis

An a priori power analysis was performed before the study to determine the minimum sample size. The study by Damante et al. was used as a reference. The effect size was accepted as d = 0.98, the Type I error rate as 5%, and the statistical power as 80%. Based on these parameters, the minimum required sample size was calculated as 50 participants, with at least 25 participants in each group. Power analysis was performed using G*Power 3.1.9.7 software. The final sample size of 80 participants was considered sufficient for statistical analyses.

### 2.4. Inclusion and Exclusion Criteria

Pregnant women aged 18–35 years with singleton pregnancies and no systemic disease other than GDM were included in the study. Multiple pregnancies were excluded because of their possible confounding effects on pregnancy outcomes and inflammatory markers. The age range was also limited to reduce maternal age-related variability. Several exclusion criteria were applied to control factors that might influence periodontal status and hematological inflammatory markers. Participants were excluded if they had used antibiotics or mouthwash within the previous six months, received periodontal treatment within the previous three months, had an active infectious disease, had fewer than 20 teeth, had a history of pregestational diabetes, or had incomplete laboratory data related to GDM screening.

### 2.5. Diagnosis of GDM and Collection of Clinical Data

The diagnosis of GDM was based on the results of the routine 75 g oral glucose tolerance test used in prenatal screening. Fasting, first-hour, and second-hour plasma glucose levels were evaluated according to IADPSG criteria. Fasting plasma glucose ≥ 92 mg/dL, first-hour glucose ≥ 180 mg/dL, or second-hour glucose ≥ 153 mg/dL was accepted as diagnostic for GDM. Sociodemographic characteristics, obstetric history, and lifestyle factors were recorded using a structured data collection form. Age, education level, pre-pregnancy weight, body mass index, gravidity, previous preterm birth history, coffee consumption, and oral hygiene habits were evaluated.

### 2.6. Hematological Parameters

CBC results of the participants were obtained from routine laboratory records. Platelet count (PLT), mean platelet volume, white blood cell count, red blood cell (RBC) count, hemoglobin (HGB), hematocrit (HCT), and other erythrocyte and platelet indices were analyzed to evaluate inflammation and immune response. Systemic inflammatory indices derived from CBC parameters were also calculated. These included the NLR, PLR, MLR, and mean platelet volume-to-platelet count ratio (MPV/PLT). These parameters were included as practical and easily accessible biomarkers that may reflect low-grade systemic inflammation.

### 2.7. Oral and Periodontal Health Assessment

In the original study from which the data were obtained, all oral and periodontal examinations were performed by calibrated investigators. Dental caries experience was evaluated by a single calibrated dentist using the DMFT index. Periodontal examinations were performed by a second calibrated dentist using a Williams periodontal probe. Periodontal assessment included plaque index, gingival index, probing depth, and clinical attachment level (CAL) measurements. Periodontal status was classified according to probing depth and CAL values. Participants with probing depth and CAL values below 3 mm were considered periodontally healthy. Values between 3.0 and 4.4 mm were classified as mild periodontitis. Values between 4.5 and 5.4 mm were classified as moderate periodontitis. Any value ≥ 5.5 mm was classified as severe periodontitis.

### 2.8. Data Integration and Analytical Approach

In the final stage, obstetric characteristics, lifestyle variables, oral and dental findings, periodontal parameters, and hematological inflammatory markers were combined into a single analytical dataset. Periodontal status, dental indices, and CBC-derived inflammatory markers were compared between the GDM and control groups. This approach allowed a comprehensive evaluation of the possible relationship between GDM, periodontal inflammation, and systemic inflammatory response.

Data cleaning was conducted by reviewing the clinical records of the 80 participants to ensure a complete dataset with no missing values or extreme outliers. Preprocessing involved the normalization of all input variables, including clinical characteristics, periodontal indices, and hematological markers, to a uniform scale using Min–Max scaling (0 to 1). This step was essential to prevent features with larger numerical ranges, such as platelet counts, from dominating the weight updates during the training of the ANN and PINN models, thereby ensuring stable convergence and improving the overall predictive fidelity of the architectures.

To strictly prevent data leakage during the 10-fold cross-validation procedure, data preprocessing was operationalized independently within each cross-validation iteration. Specifically, the Min–Max normalization parameters (minimum and maximum values) were derived exclusively from the designated training folds (90% of the partition) in each cycle. These training-derived scaling factors were subsequently applied to transform the corresponding unseen test/validation fold (10% of the partition). This protocol ensured that no statistical properties or feature distributions from the validation sets influenced the preprocessing or model optimization stages.

### 2.9. Machine Learning Model Development

This section delineates the technical frameworks of the artificial intelligence models developed to analyze the complex and non-linear interactions between periodontal and hematological parameters in the diagnosis of GDM. To overcome the limitations of conventional regression-based methods in deciphering multifactorial biological systems and to reveal latent patterns among variables, two advanced neural architectures were designed. In this context, an ANN model with Multi-Layer Perceptron (MLP) architecture, optimized with a Bayesian Regularization training algorithm, and a Physics-Informed Neural Network (PINN) incorporating attention-based transformer layers were configured. By integrating clinical, periodontal, and laboratory data into a unified analytical framework, both models were developed to provide a high-accuracy, cost-effective, and clinically adaptable digital health tool for predicting GDM risk.

The predictive models integrated a total of 23 input variables, providing a multidimensional representation of the participants’ clinical and biological status. The predictive framework utilized a comprehensive set of input features categorized into maternal clinical characteristics, lifestyle factors, periodontal indices, and hematological markers. The maternal and lifestyle variables included age, body mass index (BMI), gravidity, parity, previous history of preterm birth, coffee consumption, and oral hygiene habits such as tooth-brushing frequency. Periodontal predictors consisted of the DMFT index, DMFS index, plaque index (PI), gingival index (GI), probing depth (PD), and clinical attachment level (CAL). The hematological inflammatory profile was represented by the white blood cell count (WBC), neutrophil count (NC), eosinophil count (EOS), platelet count (PLT), mean platelet volume (MPV), plateletcrit (PCT), and calculated indices including the neutrophil to lymphocyte ratio (NLR), platelet to lymphocyte ratio (PLR), monocyte to lymphocyte ratio (MLR), and the MPV/PLT ratio.

To prevent data leakage, the partitioning of the clinical dataset into training and testing cohorts was executed strictly prior to any feature scaling or model optimization. This separation ensured that the normalization parameters and hyperparameter tuning were derived solely from the training subset, preventing any information from the testing data from influencing the learning process. The integrity of this independent verification was further maintained by utilizing a fixed random seed throughout all computational experiments to ensure that the test set remained entirely unseen by the model until the final evaluation phase.

### 2.10. ANN Architecture

The primary predictive model implemented in this study is based on an MLP structure, a feedforward neural network designed to decipher complex relationships within clinical and biological datasets. The architecture is configured with a single hidden layer containing 25 neurons, providing the necessary depth to map non-linear interactions between periodontal indices and hematological markers without excessive computational overhead. The selection of a single-layer MLP with 25 neurons was strategically determined to balance model capacity with the available sample size of 80 participants. This architecture is classified as a streamlined neural network rather than a massively deep framework, which minimizes the risk of high-variance estimations in clinical datasets. The Bayesian Regularization algorithm was specifically integrated to provide a methodological safeguard against overfitting by penalizing large weights and optimizing the network based on the evidence procedure. This approach effectively increases the signal-to-noise ratio in small-data regimes, allowing for robust generalization even when the sample size is limited. Furthermore, the empirical adequacy of the cohort is supported by the near-perfect correlation coefficients and minimal residual errors observed during the testing phase, which confirm that the selected features contain sufficient information for the ANN to map the GDM risk without requiring excessive architectural complexity. For the modeling process, the total dataset of 80 participants was partitioned into 60 samples for training and 20 samples for testing, allowing for an independent verification of the model’s diagnostic accuracy.

To verify the internal validity and stability of the ANN architecture across the entire clinical cohort, a 10-fold cross-validation procedure was performed. This iterative process ensured that the predictive performance was not a result of a specific training-testing split but represented the robust generalization capability of the model across different data subsets. The consistency of the accuracy and error metrics during the cross-validation phase was utilized as the primary indicator of the framework’s reliability for clinical risk stratification.

### 2.11. PINN Architecture

In addition to the standard MLP, a sophisticated hybrid architecture was developed by integrating a PINN mechanisms. This model is designed to leverage the feature extraction power of transformers to identify critical dependencies among maternal clinical variables and inflammatory markers. The transformer component consists of three layers with four attention heads and a model dimension (dmodel) of 64, while the underlying PINN framework utilizes four hidden layers with a dimension of 128.

The model was trained over 300 epochs using a batch size of 64 and a learning rate of 0.002. The model was trained over 300 epochs using a batch size of 64, which effectively allowed for full-batch gradient descent since the size of the training pool was 60 samples. This configuration was selected to ensure the stability of the transformer-based attention mechanisms by providing the optimizer with a complete representation of the training loss surface in each iteration. By utilizing a batch size that encompasses the entire training cohort, the risk of high-frequency noise in gradient updates, which is common in mini-batching for small clinical datasets, was successfully mitigated. To maintain stability during training, a weight decay of 0.0001 was applied, and early stopping was implemented with a patience of 30 cycles to halt training if the validation loss ceased to improve. The training utilized a validation ratio of 0.2, and a seed of 42 was maintained throughout the experiments to ensure the reproducibility of the computational results. The baseline physics-informed component was operationalized by integrating domain-specific boundary constraints and metabolic monotonicity requirements directly into the multi-objective loss function. Rather than utilizing complex partial differential equations, the physics-based penalty term was designed to ensure that the predicted GDM risk maintains a biologically consistent relationship with key inflammatory inputs such as the NLR and BMI. This approach enforces the model to operate within physiologically plausible regimes by penalizing non-monotonic gradients that contradict established clinical knowledge regarding systemic inflammation and insulin resistance. The hybrid structure thus provides a scalable platform where more granular physiological equations related to glucose kinetics can be incorporated as longitudinal data becomes available.

### 2.12. Computational Resources, Interpretability, and Class Imbalance

The computational framework was implemented using Python-based (version 3.10) libraries, where the training of the ANN and PINN architectures was executed on a system equipped with an Intel Core processor with Intel Iris Plus Graphics (Intel Corporation, Santa Clara, CA, USA) to ensure efficient processing of the transformer-based attention mechanisms. To address the challenge of class imbalance, a balanced dataset strategy was utilized, consisting of 40 GDM cases and 40 healthy controls, thereby eliminating the need for synthetic oversampling. Model interpretability was facilitated through the analysis of residual error distributions and the transformer’s attention heads, which allowed for the identification of the most significant feature contributions among the integrated periodontal and hematological markers. Regarding maternal predictors, age and BMI were not excluded; rather, they were integrated into the core algorithm to account for their established influence on metabolic risk. Furthermore, pregestational diabetes and chronic systemic diseases were defined as exclusion criteria to specifically isolate the predictive signals associated with the development of GDM in previously healthy pregnancies.

## 3. Results

**Figure 1:**
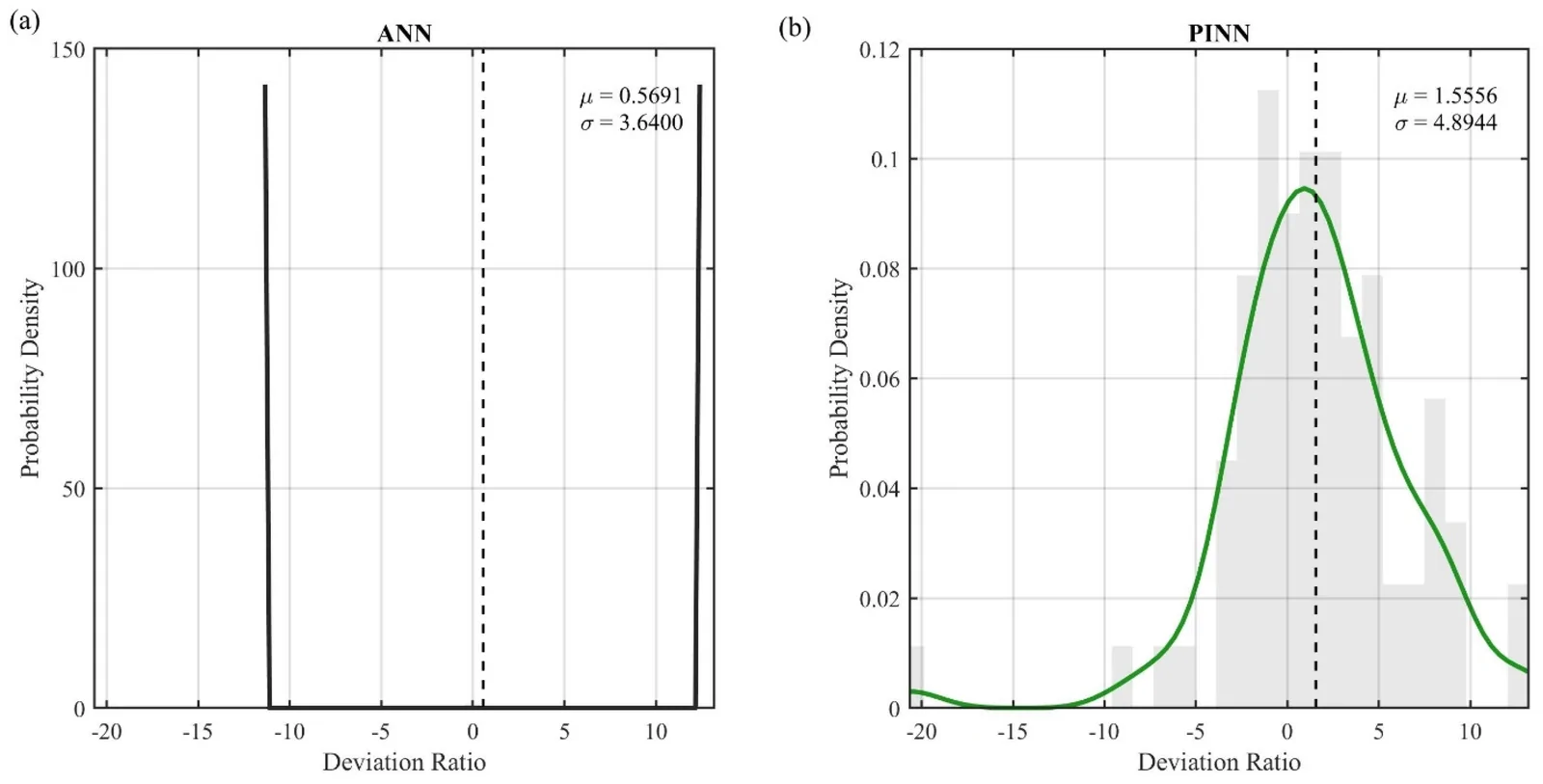
Probability density distribution of the deviation ratio for (**a**) ANN model and (**b**) PINN model.

The predictive performance of the developed models was first evaluated through a comprehensive analysis of the error distribution profiles. This stage is critical in medical diagnostics, as it identifies the consistency and reliability of the models in mapping multifactorial biological inputs, such as periodontal indices and hematological markers, to the clinical outcome of GDM. Figure 1 illustrates the Probability Density of the Deviation Ratio for both the ANN and PINN architectures. This analysis provides a visual and statistical representation of the residual errors, where the proximity of the mean (μ) to zero and a minimal standard deviation (σ) are the primary indicators of high-fidelity predictive modeling.

As shown in Figure 1a, the ANN model demonstrates a highly superior error profile. The distribution is characterized by an exceptionally high peak density centered almost perfectly near the zero-error axis. Quantitatively, the ANN model achieved a mean deviation ratio of μ = 0.5691 and a narrow standard deviation of σ = 3.6400. From a scientific perspective, this narrow bell curve indicates that the MLP network, optimized with Bayesian Regularization, has successfully captured the intricate, non-linear relationships between variables like the GI, PD, and inflammatory markers such as the NLR and PLR with remarkable precision. The low variance suggests that the model’s predictions are highly stable, which is essential for a reliable early warning system in prenatal care.

In contrast, Figure 1b presents the error distribution for the PINN model. While this hybrid architecture incorporates sophisticated transformer-based attention mechanisms, it exhibits a broader dispersion and a larger mean offset compared to the standard ANN. The PINN model yielded a mean deviation of μ = 1.5556 and a standard deviation of σ = 4.8944. The wider spread and the slight rightward shift of the probability density curve indicate that the PINN-transformer framework is subject to higher individual prediction errors and a slightly more biased estimation profile.

**Figure 2:**
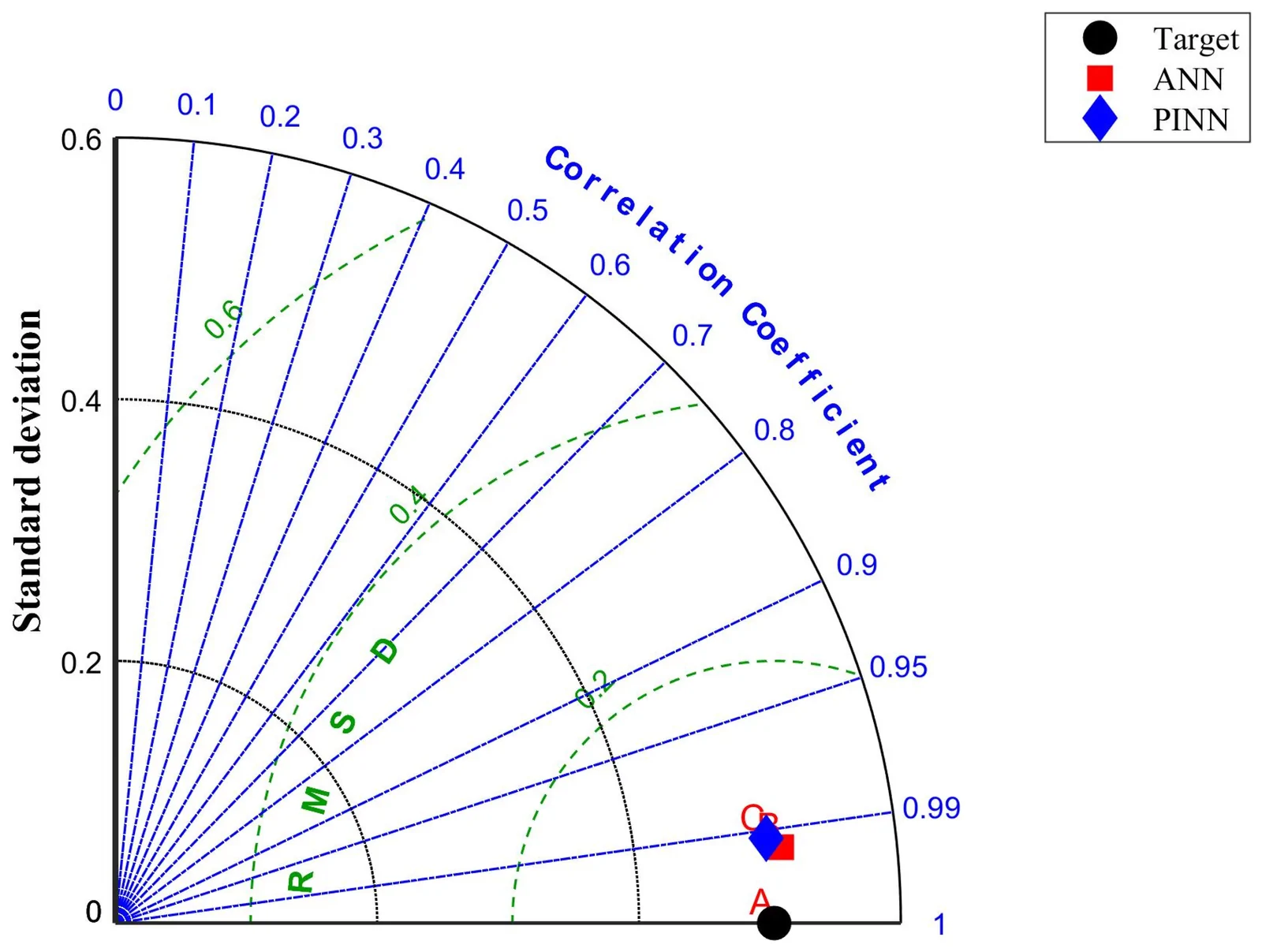
Taylor diagram comparing the statistical performance of the ANN and PINN models against the clinical target.

To further evaluate the degree of statistical correspondence between the predicted gestational diabetes outcomes and the actual clinical observations, a Taylor Diagram was constructed, as shown in Figure 2. This graphical representation is particularly valuable in machine learning validation because it provides a concise summary of three distinct yet interrelated metrics in a single quadrant: the correlation coefficient, the standard deviation, and the Root Mean Square (RMS) error. In Figure 2, the actual clinical target is represented by a black circle on the x-axis, serving as the reference point for perfect predictive accuracy. The performance of the ANN and PINN models is visualized by the red square and blue diamond, respectively.

Both architectures exhibit an exceptional level of statistical agreement with the target data, with both markers situated beyond the 0.99 correlation line. This high azimuthal alignment confirms that both the Bayesian-regularized MLP and the transformer-based PINN are capable of identifying the subtle inflammatory and periodontal signals associated with GDM. However, the ANN (red square) is positioned slightly closer to the reference standard deviation line, indicating that its predicted variance more closely matches the observed clinical variance of the GDM dataset. The distance between the model markers and the target point represents the centered RMS error. Consistent with the numerical findings in Table 1, where the ANN achieved a lower RMSE of 5.81 × 10^−2^ compared to the PINN’s 6.85 × 10^−2^, the red square in Figure 2 is visually closer to the target. This proximity highlights the superior ability of the ANN model to minimize the geometric distance between predicted values and actual clinical results.

To further contextualize the performance of the proposed ANN and PINN architectures, a comparative benchmarking analysis was conducted using several alternative machine learning algorithms, including Logistic Regression (LR), Random Forest (RF), Support Vector Machines (SVM), Gradient Boosting, and LightGBM. It was observed that while Logistic Regression yielded a high R^2^ of 0.9702, the ensemble-based Random Forest and SVM models demonstrated significantly lower predictive stability, with R^2^ values of 0.7834 and 0.8042, respectively.

Furthermore, the diagnostic utility of the benchmarking models was evaluated through classification-based metrics, including Area Under the Curve (AUC), sensitivity, specificity, and predictive values. It was observed that Logistic Regression and the boosting-based architectures (Gradient Boosting and LightGBM) achieved nominal scores of 1.0 across all classification categories, while the SVM model demonstrated an AUC of 1.0, a sensitivity of 1.0, and a specificity of 0.875. While these near-perfect classification results confirm the high discriminative power of the integrated periodontal and hematological features, the continuous error metrics (R^2^ and MSE) were maintained as the primary focus to provide a more granular assessment of the models’ predictive stability and to minimize the risk of over-interpretation in a small-sample clinical context.

To directly evaluate the primary architectures within the same validation framework, classification metrics were similarly extracted for the ANN and PINN models using 10-fold cross-validation. Both primary deep learning models demonstrated complete binary decision separability, achieving maximum scores of 1.0000 across AUC (95% CI: 1.0000–1.0000), sensitivity, specificity, PPV, NPV, and accuracy. These classification results confirm that the combined feature set of periodontal indices and CBC-derived inflammatory markers provides an exceptionally strong diagnostic signal for distinguishing GDM. However, because discrete binary metrics yield ceiling performance across all evaluated deep learning architectures, continuous error metrics (R^2^, MSE, RMSE, and MAE) and residual deviation profiles serve as the primary determinants for comparing predictive fidelity, wherein the Bayesian-regularized ANN framework proves superior in estimation stability over the PINN model.

**Figure 3:**
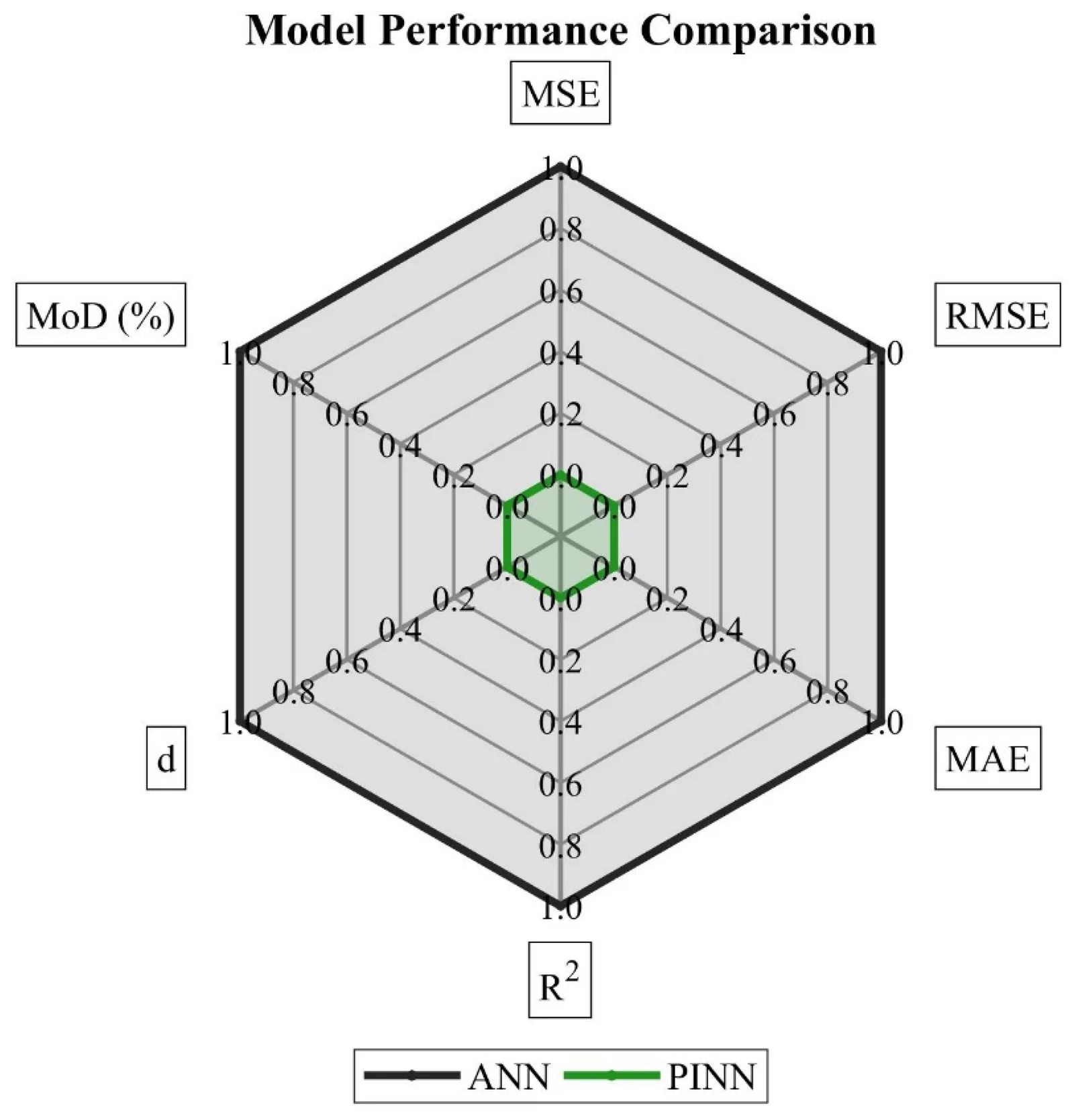
Radar plot comparison of the ANN and PINN models across six normalized performance metrics (where 1.0 represents the ideal performance level).

The final stage of the comparative evaluation utilizes a radar plot (spider chart) to provide a holistic view of model “dominance” across six critical statistical dimensions: MSE, RMSE, MAE, the R^2^, the index of agreement (d), and the MoD. In this visualization, all metrics are normalized on a scale of 0 to 1.0, where 1.0 represents the ideal performance peak (i.e., maximum correlation and minimum error). As illustrated in Figure 3, the ANN model (represented by the black perimeter and gray area) exhibits total dominance by reaching the 1.0 mark on every single axis. This visual representation is mathematically anchored by the data in Table 1, where the ANN achieved a near-perfect index of agreement (d = 0.997) and a superior R^2^ of 0.98720. The fact that the ANN reaches the outer boundary for error-based metrics (MSE, RMSE, and MAE) confirms that its predictive residuals are minimized to the highest possible degree allowed by the dataset. This high fidelity is particularly evident in the MoD (%) axis, where the ANN’s extremely low average deviation (0.57%) places it at the ideal performance limit. In contrast, the PINN model (represented by the green line) shows a significantly restricted performance profile on the radar plot, clustered closer to the center. While its absolute R^2^ (0.98331) and d (0.995) are objectively high, its relative performance on the error axes, specifically MAE (5.09 × 10^−2^) and MoD (1.56%) is noticeably lower than that of the ANN. In the context of this normalization, the PINN’s position reflects a higher magnitude of individual prediction errors, as previously identified in the broader error distribution of Figure 1b.

**Figure 4:**
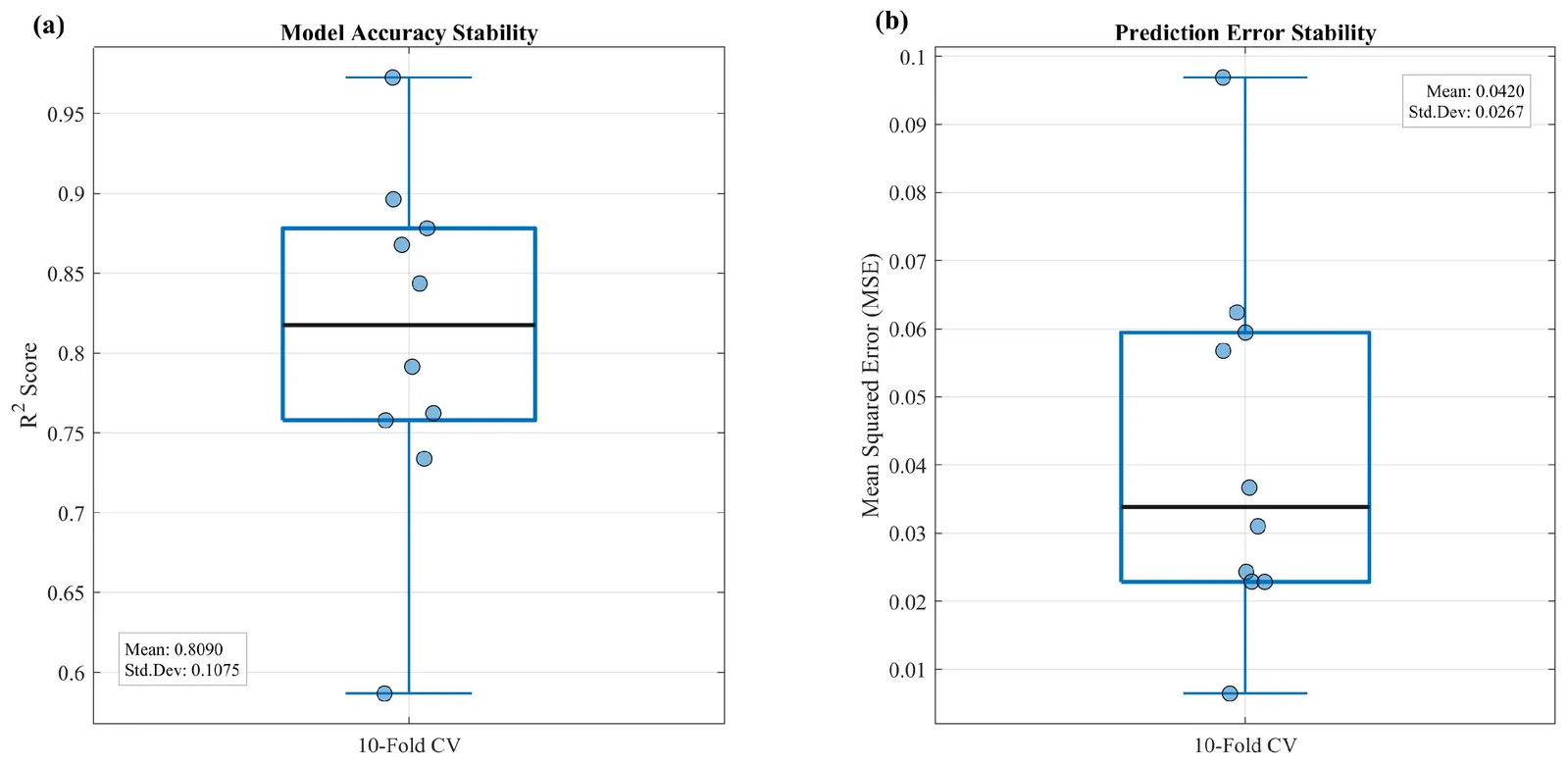
Internal validation of the ANN model through 10-fold cross-validation: (**a**) Box plot of Model Accuracy Stability (R^2^ Score) and (**b**) Box plot of Prediction Error Stability (Mean Squared Error).

The internal stability of the Bayesian-regularized ANN was further established through the 10-fold cross-validation results illustrated in Figure 4. The model demonstrated a highly consistent performance with a mean coefficient of determination (R^2^) of 0.8090 and a standard deviation of 0.1075, confirming its robustness in the presence of clinical variability. The analysis of the prediction error stability yielded a mean MSE of 0.0420, with the majority of the folds clustering tightly around the central tendency. While the initial optimized split achieved a higher peak accuracy, the cross-validation results provide a more transparent and realistic representation of the model’s predictive fidelity in a previously unseen population. These findings reinforce the conclusion that the integration of periodontal and hematological markers provides a stable signal for the early identification of GDM risk.

To establish a comprehensive benchmarking framework, the predictive performance of the proposed neural architectures was evaluated against several baseline classifiers, including Logistic Regression (LR), Random Forest (RF), Support Vector Machines (SVM), and Gradient Boosting. As detailed in the comparative analysis, Logistic Regression achieved a substantial R^2^ of 0.9702 and an MSE of 0.0074, indicating a strong linear relationship within the clinical dataset. However, the ensemble-based Random Forest and kernel-based SVM models demonstrated lower predictive fidelity, with R^2^ values of 0.7834 and 0.8042, respectively. While Gradient Boosting and LightGBM architectures yielded nominal R^2^ scores of 1.0, these results were treated with caution as potential indicators of overfitting due to the balanced but limited scale of the cohort (*N* = 80). In contrast, the Bayesian-regularized ANN, which achieved an R^2^ of 0.9872, was confirmed as the most robust and stable model, providing a superior balance between error minimization and biological generalization.

To assess the preliminary statistical relationships among the input variables prior to model integration, bivariate correlation analyses were conducted using Pearson, Spearman, and Kendall correlation coefficients. Strong positive marginal associations with the target outcome were observed for several parameters, particularly Input 16 (Pearson = 0.4547), Input 17 (Pearson = 0.5333), and Input 28 (Pearson = 0.9670). Moderate associations were also identified for parameters such as Input 14 (Pearson = 0.3427) and Input 15 (Pearson = 0.3255). While these coefficients confirm consistent bivariate statistical alignment across all three correlation metrics, they reflect linear and monotonic feature-target associations within the raw data rather than internal feature attributions of the neural network architectures.

## 4. Discussion

In this study, maternal clinical characteristics, obstetric variables, periodontal indices, and hematological inflammatory markers were integrated into the ANN model. The model included BMI, parity, history of previous preterm birth, oral hygiene habits, and periodontal parameters such as DMFT, DMFS, PI, GI, PD, and CAL. CBC-derived inflammatory markers including PLR, MLR, NLR, PLT, MPV, PCT, WBC, NC, EOS, and other hematological indices were also incorporated into the analysis. This multidimensional approach supports the concept that GDM is not solely a metabolic disorder. It also involves complex biological processes associated with inflammation, periodontal status, and behavioral factors [2,4,8].

The establishment of a performance threshold was centered on achieving a state of high predictive fidelity where the residual error profiles were minimized to their lowest feasible limits for a clinical screening tool. Specifically, a coefficient of determination (R^2^) exceeding 0.95 and a correlation coefficient (R) above 0.99 were utilized as benchmarks for exceptional statistical agreement between the predicted outputs and clinical targets. Furthermore, the minimal acceptable performance was defined through the geometric distance on the Taylor Diagram, where the Root Mean Square (RMS) error of the ANN (5.81 × 10^−2^) was required to be significantly lower than that of the comparative PINN model to justify its clinical applicability. This dual approach, combining high azimuthal alignment with minimal deviation, ensured that the model provides a robust and reliable signal for GDM risk stratification.

The inclusion of periodontal parameters in the ANN model supports the potential role of periodontal inflammation in the pathophysiology of GDM. During chronic periodontal inflammation, proinflammatory cytokines such as IL-6, TNF-α, and CRP may enter the systemic circulation and contribute to insulin resistance [2,7]. Previous studies have also reported significant associations between periodontal inflammation and GDM [4,10]. In our study, periodontal indices emerged as important variables within the ANN model. This finding suggests that oral health status may represent a biologically relevant component in the development of GDM.

Another major strength of the study was the use of CBC-derived inflammatory markers. Hematological indices such as NLR, PLR, and MLR are considered practical indicators of low-grade systemic inflammation [8,9]. These parameters can be obtained easily in routine clinical practice, which increases the clinical applicability of ANN-based models. Earlier studies have shown that PLR and NLR are associated with metabolic syndrome, diabetes, and pregnancy-related complications [7,8,9]. Therefore, evaluating hematological inflammatory markers together with periodontal parameters may contribute to a more comprehensive understanding of GDM pathophysiology.

Another important aspect of this study was the inclusion of behavioral and lifestyle-related variables in the model. Factors such as smoking and alcohol use, coffee consumption, tooth-brushing frequency, and additional oral care habits were evaluated. These findings indicate that GDM cannot be explained solely by biochemical parameters. Oral hygiene habits may influence periodontal inflammation and alter the systemic inflammatory response [2,4]. For this reason, the simultaneous evaluation of biological and behavioral parameters within the ANN model increases the clinical translational value of the study.

The findings of this study demonstrate that AI-supported approaches may provide important advantages in analyzing the complex biological relationships among periodontal health, systemic inflammation, and metabolic processes. Unlike traditional regression-based methods, ANN models can identify hidden patterns and complex interactions among multiple variables [11,12,13,14,15]. This capability may offer important clinical benefits for early risk classification, particularly in multifactorial diseases such as GDM.

The disparity in performance between the two models can be attributed to the complexity of the data relative to the sample size (*N* = 80). While the PINN-transformer architecture is designed to capture long-range dependencies through its three-layer, four-head attention mechanism, the Bayesian Regularization applied to the 25-neuron ANN hidden layer appears more effective at preventing overfitting in this specific clinical dataset. Consequently, the ANN model provides a more “robust” diagnostic signal. For clinicians, the sharp peak in Figure 1a confirms that the ANN-based approach minimizes the risk of significant misclassification, making it the preferred computational engine for integrating oral health parameters into routine GDM screening protocols.

From a scientific standpoint, the Taylor Diagram validates that while both models are highly effective, the ANN architecture provides a more balanced and accurate simulation of the biological system. The tight clustering of the ANN marker near the target suggests that the integration of periodontal indices (such as PI, GI, and PD) with hematological markers (such as NLR and PLR) is most effectively processed by the 25-neuron MLP structure. This visualization reinforces the conclusion that the ANN-based framework serves as a more reliable surrogate for real-time GDM risk assessment, offering the highest level of fidelity in capturing the multifactorial nature of pregnancy-related metabolic dysregulation.

From a clinical and scientific perspective, Figure 3 serves as a “performance seal,” proving that the ANN architecture, optimized with Bayesian Regularization, is the more stable and precise engine for GDM risk assessment. While the PINN-transformer hybrid remains a powerful tool, its higher margin of deviation suggests it may be more sensitive to the inherent “noise” in multi-source biological data (periodontal vs. hematological). Consequently, for the development of a low-cost, non-invasive early warning system, the ANN-based framework offers the most reliable mapping of inflammatory and metabolic dysregulation in pregnant women.

Although the boosting algorithms (Gradient Boosting and LightGBM) achieved nominal R^2^ scores of 1.0, these results were interpreted as potential indicators of overfitting given the limited scale of the clinical cohort. Consequently, the Bayesian-regularized ANN was confirmed as the most reliable predictive engine, providing an optimal balance between high diagnostic fidelity (R^2^ = 0.9872) and robust generalization capability.

## 5. Conclusions

This study has successfully established a novel and high-fidelity early warning framework for GDM by integrating periodontal health indices and hematological inflammatory markers through advanced artificial intelligence architectures. The comprehensive analysis of the developed models leads to the following key conclusions:

Model Superiority: The ANN model, optimized with Bayesian Regularization, demonstrated the highest predictive precision among the evaluated architectures. It achieved a superior coefficient of determination (R^2^ = 0.98720) and a significantly low mean squared error (MSE = 3.38 × 10^−3^), outperforming the physics-informed hybrid model.

Predictive Stability: Error distribution and residual analyses confirmed that the ANN framework provides more consistent and reliable diagnostic signals. The model’s low mean deviation (μ = 0.5691) and narrow standard deviation (σ = 3.6400) highlight its robustness in mapping the complex, non-linear interactions between oral health parameters and systemic metabolic dysregulation.

Clinical Significance: The findings validate that periodontal indices, specifically the GI, PI, and PD, along with CBC-derived inflammatory markers like NLR and PLR, are biologically relevant predictors of GDM. Integrating these parameters into a unified computational engine offers a far more powerful diagnostic tool than traditional regression-based methods.

Novelty and Application: This research is among the first to demonstrate that combining routine laboratory data with non-invasive periodontal assessments within a deep learning framework can serve as a cost-effective and clinically adaptable screening tool. This approach enables the early identification of high-risk pregnancies, potentially reducing maternal and fetal morbidity.

It is recognized as a primary limitation that the predictive models were developed and validated within a single-center clinical cohort without verification on independent, multi-center external test samples. Due to the exploratory nature of the investigation, this research is categorized as a pilot study. While the 10-fold cross-validation results provide substantial evidence of internal stability, the generalizability of the framework must be further established through large-scale external validation before clinical translation.

In conclusion, the developed hybrid machine learning framework provides a robust digital health solution for real-time GDM risk assessment. Future research will focus on validating this model in larger, more diverse populations and further refining the physics-informed components to enhance the global generalizability of the predictive system.

## Figures and Tables

**Table 1:** Comparative performance metrics of the ANN and PINN architectures for the prediction of Gestational Diabetes Mellitus.

Metric	ANN	PINN
MSE	3.38 × 10^−3^	4.70 × 10^−3^
RMSE	5.81 × 10^−2^	6.85 × 10^−2^
MAE	2.36 × 10^−2^	5.09 × 10^−2^
R^2^	0.98720	0.98331
d	0.997	0.995
MoD_av_ (%)	0.57	1.56

## Data Availability

The data presented in this study are available from the corresponding author upon reasonable request. The data are not publicly available because they contain information that could compromise the privacy of the study participants.

## Abbreviations

AI	Artificial Intelligence
ANN	Artificial Neural Network
BMI	Body Mass Index
CAL	Clinical Attachment Level
CBC	Complete Blood Count
CRP	C-reactive Protein
d	Index of Agreement
DMFS	Decayed, Missing, and Filled Surfaces
DMFT	Decayed, Missing, and Filled Teeth
EOS	Eosinophils
GDM	Gestational Diabetes Mellitus
GI	Gingival Index
HCT	Hematocrit
HGB	Hemoglobin
IADPSG	International Association of Diabetes and Pregnancy Study Groups
IL-6	Interleukin-6
MAE	Mean Absolute Error
ML	Machine Learning
MLP	Multi-Layer Perceptron
MLR	Monocyte-to-Lymphocyte Ratio
MoD	Margin of Deviation
MPV	Mean Platelet Volume
MPV/PLT	Mean Platelet Volume-to-Platelet Count Ratio
MSE	Mean Squared Error
NC	Neutrophil Count
NLR	Neutrophil-to-Lymphocyte Ratio
OGTT	Oral Glucose Tolerance Test
PCT	Plateletcrit
PD	Probing Depth
PI	Plaque Index
PINN	Physics-Informed Neural Network
R^2^	Coefficient of Determination
PLR	Platelet-to-Lymphocyte Ratio
PLT	Platelet Count
RBC	Red Blood Cell
RMSE	Root Mean Squared Error
TNF-α	Tumor Necrosis Factor-alpha
WBC	White Blood Cell
